# Macrophage volume analysis and real-time monitoring by digital holography

**DOI:** 10.1117/1.JBO.31.6.066002

**Published:** 2026-06-03

**Authors:** Baodan Bai, Yanwei Lu, Geng Zhu, Feiyu Liu, Yubo Liu, Wen Wu, Dezhi Lu, Xiupin Wu

**Affiliations:** a Shanghai University of Medicine and Health Sciences, School of Medical Instruments, Innovation Center for Intelligent Ophthalmic Technologies and Equipments, Shanghai, China; b Shanghai University of Science and Technology, School of Health Science and Engineering, Shanghai, China; c Chongqing Medical and Pharmaceutical College, Chongqing Engineering Research Center of Pharmaceutical Sciences, Chongqing, China; d Shanghai Ninth People’s Hospital, Shanghai Jiao Tong University School of Medicine, Department of Orthopaedic Surgery, Shanghai Key Laboratory of Orthopaedic Implants, Shanghai, China

**Keywords:** label-free measurement of cell volume, volume monitoring, 3D morphology of macrophages, digital holographic microscopy

## Abstract

**Significance:**

Cell volume serves as a macroscopic indicator of cellular metabolism, growth, and signal transduction. Understanding how cell volume varies across macrophage phenotypes is essential for interpreting their functional states and activation processes.

**Aim:**

We present a label-free, noninvasive method for 3D morphological characterization and volume quantification of macrophages and apply it to monitor volume variations during their biological behaviors.

**Approach:**

We employed a home-made off-axis digital holographic microscopy (DHM) system based on a Mach–Zehnder interferometric configuration to reconstruct the 3D morphology of cells. Using the mathematical relationship between the phase map and the cell thickness, we further calculated their cellular volume.

**Results:**

Our measurements show that M0 macrophages have a nearly spherical morphology with a volume of $970.54\pm 153.15\;\mu m^{3}$. M1 macrophages exhibit a flattened, pancake-like morphology with pseudopodia, with a significantly larger volume of $3822.00\pm 437.45\;\mu m^{3}$. M2 macrophages present a morphology resembling that of M0 cells but with a volume of $2743.10\pm 254.67\;\mu m^{3}$. It indicates that the cell volume might be a potential parameter to distinguish different polarized macrophages. In addition, we successfully monitored the volume changes of macrophages during cell death, cell division, and the polarization process from M0 to M1. The cell-death process behaved as expected, with the cell volume continuously decreasing and eventually reaching a plateau. The division event we observed occurred together with cell death; during division, the total volume of the cell cluster increased sharply and then gradually decreased as cell death progressed. During the 8-h M0-to-M1 polarization process, two phases of gradual volume increase and two phases of rapid volume increase were observed. In the early stage (0 to 2 h), M0 macrophages were likely not fully activated, showing only mild volume changes but still exhibiting an increasing trend. At 6 to 8 h, the cells undergo a sharp increase in volume, reflecting their full activation and transition toward the M1 phenotype.

**Conclusions:**

We demonstrate that DHM offers a powerful label-free strategy for quantifying macrophage volume and capturing the volume dynamic. It might be a potential approach for a macroscopic characterization to observe intracellular activities.

## 1 Introduction

Digital holographic microscopy (DHM) is an interferometric imaging technique capable of recording both amplitude and phase information of optical wavefronts.^1^^–^^3^ It is a label-free and noninvasive quantitative phase imaging technology. Based on the optical configuration, DHM can be classified into on-axis and off-axis modalities. In on-axis DHM, the object and reference beams propagate along the same optical axis, which simplifies the optical setup but leads to the superposition of zero-order, twin-image, and real-image terms in the reconstruction process.^4^ By contrast, off-axis DHM introduces a small angular tilt between the object and reference beams, improving reconstruction efficiency and temporal resolution.^5^ It is more suitable for investigating cellular biophysical properties and functional states.

Macrophages play a crucial role in the immune system by forming the initial line of defense through the release of pro-inflammatory cytokines, helping to activate the innate immune system as well as subsequent T and B cell responses.^6^^–^^8^ Macrophages exhibit three phenotypes: M0, M1, and M2. Upon external stimulation, M0 macrophages can differentiate into either M1 (pro-inflammatory) or M2 (anti-inflammatory) phenotypes. Under inflammatory conditions, external pharmacological treatment can induce the transition of macrophages from the M1 to the M2 phenotype.^9^ These processes play vital roles in wound healing and tissue remodeling.^10^

Research shows that macrophage volume might regulate phenotypic switching by signaling pathways and gene expression. Yang et al. realized the phenotypic polarization of macrophages from M1 to M2 by regulating PEG concentration, which induced a reduction in macrophage volume mediated by the JAK/STAT signaling pathway.^11^ Jiao et al. regulated macrophage volume to promote macrophage polarization toward the M1 phenotype through nuclear envelope proteins SUN1/2, under the influence of inflammatory signals such as lipopolysaccharide (LPS), resulting in enhanced inflammation and anti-tumor immunity.^12^ These results imply that the macrophage volume is an important feature during phenotypic transition, and the observation of its dynamic changes plays a crucial role in the regulation of macrophage function.

Owing to its label-free and noninvasive nature, DHM has been adopted to measure the morphology, mass, and density changes in response to external stimuli without perturbing cellular function. Previous studies have shown that macrophage dry mass changes measured by DHM provide a highly sensitive indicator of cellular responses to nanomaterial exposure, enabling earlier detection of cytotoxic effects than conventional biochemical assays.^13^^–^^15^ In infection models, the healthy and infected macrophages could be discriminated by macrophage swelling and refractive index variations associated with phagocytosis and intracellular pathogen accommodation.^16^ Recent developments in phase-based imaging have enabled 3D and time-resolved tracking of macrophage apoptosis and cell division, providing insight into dynamic immune-cell behaviors at the single-cell level.^17^^–^^18^ However, most studies focus on dry mass or qualitative morphological changes, whereas quantitative investigations of volumetric dynamics and their functional relevance to macrophage activation and polarization remain limited.

This work focuses on the 3D morphology and volume of macrophages. Using DHM, we quantitatively measured macrophage volume and analyzed volumetric and morphological differences among M0, M1, and M2 polarization states. Furthermore, we monitored the volume dynamics during M0 macrophage cell death, M1 macrophage division, and the polarization transition from M0 to M1. The measurement of the cell volume and derived dynamic parameters—such as the slope of volumetric change—might provide a new perspective for investigating functional activities and underlying mechanisms in living cells.

## 2 Materials and Methods

### 2.1 Macrophage Volume Measurement Method

Digital holography consists of two processes, interference recording and diffraction reconstruction. Both amplitude and phase information of the object is recorded by the photodetector, and then, it is restored based on the light diffraction principle. The intensity distribution of the hologram incident on the sensor plane is given by^16^

$$I(x,y)=|O(x,y)+R(x,y){|}^{2}=|O{|}^{2}+|R{|}^{2}+OR^{*}+RO^{*},$$ (1)

where * represents complex conjugation. In the equation, $|O{|}^{2}$ and $|R{|}^{2}$ are the object wave and reference wave, respectively, and ${OR}^{*}$ and ${RO}^{*}$ denote the object image and the conjugate image, respectively.

For high-quality reconstruction, except ${OR}^{*}$, the other three terms should be filtered through a low-pass filter in Fourier space first. Then, the object light field could be reconstructed by the angular spectrum algorithm,

$$U(x_{i},y_{i})=F^{-1}\{F[R(x,y)I(x,y)]\cdot \exp[j\frac{2\pi }{\lambda }d_{i}\sqrt{\left(1-(\lambda f_{x}{)}^{2}-{\left(\lambda f_{y}\right)}^{2}\right)}]\},$$ (2)

where $\exp[j\frac{2\pi }{\lambda }d_{i}\sqrt{\left(1-(\lambda f_{x}{)}^{2}-{\left(\lambda f_{y}\right)}^{2}\right)}]$ is the transfer function of the angular spectrum method, λ denotes the wavelength, and $d_{i}$ is the reconstruction distance.

The intensity $I(x_{i},y_{i})$ and phase distribution $\theta (x_{i},y_{i})$ of the object could be calculated by

$$I(x_{i},y_{i})=|U(x_{i},y_{i}){|}^{2},$$ (3)

θ(xi,yi)=arctan{ImU(xi,yi)ReU(xi,yi)}, (4)

where Im represents the imaginary part symbol and Re represents the real part symbol.

Then, the cell volume, cell circularity, and sphericity could be measured using the unwrapped phase Δθ(x,y), with distortion correction. By integrating the cell thickness map, the cell volume can be calculated by

V=∫∫h(x,y)dxdy, (5)

where $h(x,y)=\frac{\lambda }{2\pi [\Delta n-n_{0}]}\Delta \theta (x,y)$, Δn is the refractive index of the target object, and $n_{0}$ is the refractive index of the surrounding medium. To estimate the cell thickness and subsequently the volume, we assumed a constant average refractive index for the macrophages ($n_{\mathrm{cell}}=1.38$) and the surrounding culture medium ($n_{0}=1.33$) based on typical mammalian cell values. Note that because the exact intracellular refractive index distribution is coupled with the cell thickness in the phase measurement, the cell volume calculated here represents the effective optical volume.

### 2.2 Macrophage Culture

RAW 264.7 macrophages were cultured in 1640 medium (Wuhan Saiwei Biotechnology, China) supplemented with 10% fetal bovine serum (Wuhan Saiwei Biotechnology, China) and passaged at a 1:3 ratio upon reaching 80% to 90% confluency. To obtain M0 macrophages, well-grown RAW264.7 cells were centrifuged at 1000 rpm for 5 min(min) at 37°C. The supernatant was discarded, and the cell pellet was resuspended and seeded into culture dishes. After incubation at 37°C for 2 to 3 h, nonadherent cells were washed away, and the remaining adherent cells were considered M0 macrophages. M1 macrophages were generated by treating M0 cells with 200 ng/mL of LPS, followed by incubation at 37°C in a 5% ${\mathrm{CO}}_{2}$ incubator for 24 h. Similarly, M2 macrophages were induced by exposing M0 cells to 20 ng/mL of IL-4 under the same incubation conditions for 24 h.

We fix the cells with 4% paraformaldehyde (Wuhan Saiwei Biotechnology, China) for 10 to 20 min and then treat them with 0.5% Triton X-100 for 5 min. We dilute and add fluorescently labeled phalloidin to the cells, followed by incubation at room temperature in the dark for 20 to 30 min.

## 3 Experiments and Results

### 3.1 DHM Setup

**Fig. 1:**
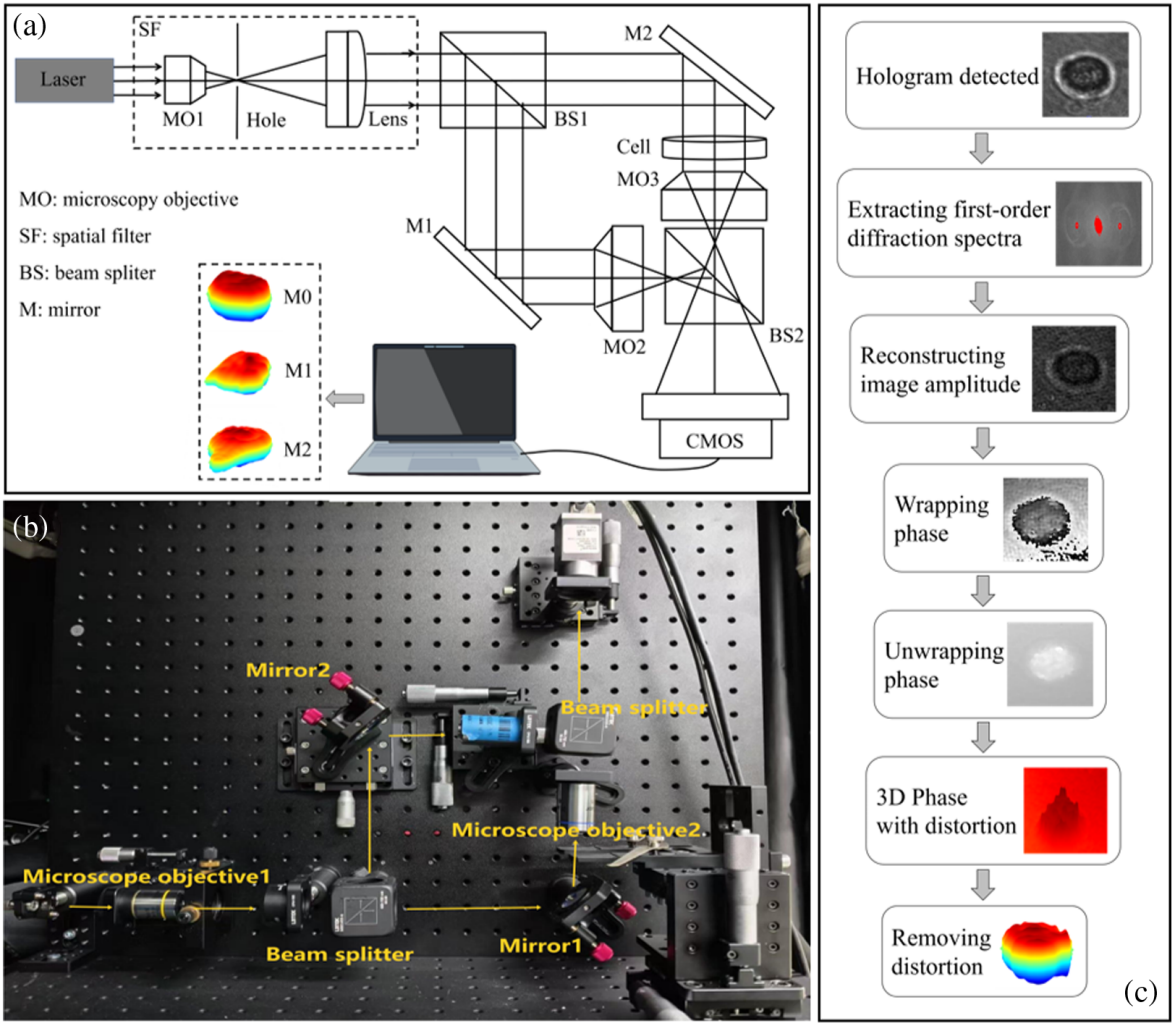
(a) Schematic illustration of DHM-based macrophage imaging process. (b) Home-made DHM system. (c) 3D reconstruction procedure for the cell.

The DHM system was set up based on a Mach–Zehnder interferometer, and its schematic is displayed in Fig. 1(a). The light emitted from the laser (MDL-III-633 nm-30 mW, Changchun New Industries Optoelectronics Technology Co., Ltd.) passes through a spatial filter for removing the stray light and collimated beam expansion and is then split into two beams by the beam splitter BS1. One beam was reflected by mirror M1 and magnified by the microscope objective (MO2, [60×,NA=0.75]), serving as the reference beam. The other beam passed through mirror M2, transmitted through the sample, and was subsequently magnified by another identical microscope objective (MO3), forming the object beam. During the numerical reconstruction process, the precise reconstruction distance was automatically determined by evaluating the frequency energy of the reconstructed amplitude image to ensure optimal focus. The two beams were then recombined at the beam splitter (BS2) and detected by the CMOS with the pixel size of 3.45  μm×3.45  μm (acA2440-20gm, Basler).

We followed the workflow shown in Fig. 1(c) to process the holograms acquired by the home-made system in Fig. 1(b) and to recover the 3D morphology of the cells. First, we obtain the Fourier spectra and locate the centers and spectral ranges of the zero-order and the ±1st-order diffraction terms. After removing the zero-order and −1st-order interference components, the hologram only including the +1st-order is restored, and numerical reconstruction is performed to obtain the wrapped phase information. The wrapped phase is constrained within the range of −π to π, resulting in phase discontinuities. In this case, a quality-guided phase unwrapping algorithm is required to further recover the unwrapped phase and obtain the smooth 3D morphological distribution of the cells.

In 3D phase reconstruction, phase unwrapping, distortion removal, and cell contour extraction are crucial for accurate volume quantification. First, a quality-guided phase unwrapping algorithm was utilized to recover the continuous phase.^19^ By evaluating the phase quality metric of each pixel, we identify potential phase discontinuities (phase jumps). When a discontinuity point is detected, an integer multiple of 2π is added or subtracted to restore phase smoothness, whereas the remaining pixels retain their original phase values. Next, to remove phase distortions, a global compensation using Zernike polynomial fitting combined with a local compensation via median filtering was applied to yield a flat background. The Zernike polynomial fitting method is applied to estimate and subtract the global background phase curvature.^20^ However, as the ideal Zernike polynomials may not perfectly capture all complex optical aberrations, residual local background phase distortions frequently remain. In this case, a median filtering approach might be applied to yield a clean background phase distribution. Finally, a segmentation method based on dual-thresholding combined with morphological reconstruction was employed.^21^ Specifically, high- and low-threshold masks were generated based on the physical thickness map. The high-threshold mask is utilized to identify the thickest central core region of the cell, whereas the very low-threshold mask (i.e., the boundary mask) is designed to capture the entire spatial extent of the cell, including delicate pseudopodia, along with potential background noise. The morphological reconstruction is then performed using the high-threshold mask as the seed for connected-component growing. In addition, an area opening operation is applied to remove any residual tiny isolated noise pixels, followed by a morphological closing operation to fill potential internal micro-holes, ultimately yielding the precise cell segmentation mask.

To independently validate the volumetric accuracy of our DHM system, we imaged standard monodisperse silica microspheres (diameter: 10  μm, refractive index: 1.46) suspended in the phosphate-buffered saline (PBS) solution (refractive index: 1.34). Based on the standard geometric formula $V=4\pi r^{3}/3$, the theoretical geometric volume is $523.60\;\mu m^{3}$. Using our DHM system, the measured volume of N=15 individual microspheres was $531.47\pm 65.35\;\mu m^{3}$, with the average relative error of ∼1.5%.

### 3.2 Volume Measurements of Macrophages in Three Polarization States

**Fig. 2:**
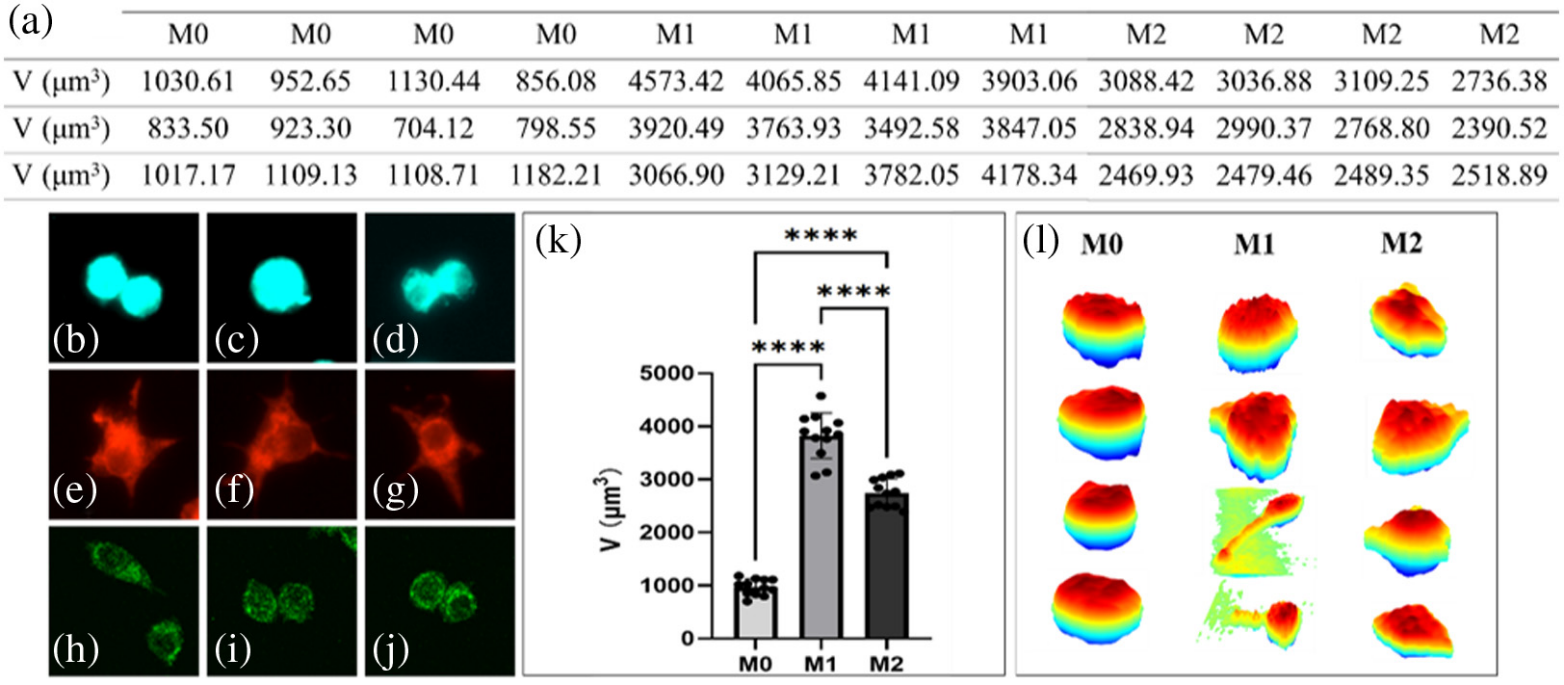
Morphological distributions and volume statistics of macrophages. (a) Volumes of M0, M1, and M2 (N=12 cells for each phenotype, total N=36). (b)–(d), (e)–(g), and (h)–(j) Florescent images of M0, M1, and M2, respectively. (k) Statistic results for the volumes. (l) 3D morphological distributions measured by our DHM system.

Experiments were conducted using macrophages with three different polarization phenotypes. We incubate the cells with iNOS primary antibody/CD206 primary antibody at 4°C overnight in the dark, wash with PBS, and then incubate with fluorescent secondary antibody at room temperature in the dark for 60 to 90 min. Then, specific proteins were detected via fluorescence microscopy, and imaging was performed using label-free DHM. Figures 2(b)–2(j) show macrophages of different phenotypes under fluorescence microscopy. Figures 2(b)–2(d) display the morphology of M0 macrophages observed under fluorescence microscopy by labeling cytoskeletal proteins. Figures 2(e)–2(g) show the morphology of M1 macrophages observed under fluorescence microscopy by labeling iNOS proteins. Figures 2(h)–2(j) depict the morphology of M2 macrophages observed under fluorescence microscopy by labeling CD206 proteins.

We then performed label-free, noninvasive 3D morphological measurements and volume statistics of macrophages with known polarization states using the DHM system. Representative volume values are shown in Fig. 2(a). The volumes of M0 cells ranged from 704.12 to $1182.21\;\mu m^{3}$, with an average of $970.54\;\mu m^{3}$ and a variance of $153.15\;\mu m^{3}$. In comparison, the volume distributions of M1 and M2 macrophages were $3822.00\pm 437.45\;\mu m^{3}$ and $2743.10\pm 254.67\;\mu m^{3}$, respectively. Based on the mean values, M0 cells exhibited the smallest volume; upon polarization into the M1 phenotype, the volume increased markedly and then decreased again after transitioning to the M2 phenotype, although it remained larger than that of M0 cells. In terms of volume fluctuations, all three types showed variations within ∼10% of their respective mean values.

To further investigate the volume variation trends among the three polarized macrophage phenotypes, we employed GraphPad Prism 10 to plot the histograms of M0, M1, and M2 macrophages, as shown in Fig. 2(k). It can be found that the cell volume shows a sharp increase from the M0 to the M1 state, followed by a moderate decrease upon transitioning to the M2 phenotype. Figure 2(l) presents the 3D morphological distributions of the three macrophage types; the first to third columns correspond to the M0, M1, and M2, respectively. M0 cells exhibit a smooth and nearly spherical morphology, appearing almost circular in the top view. By contrast, M1 macrophages display enlarged cell bodies or extensions in multiple directions, with irregular, undulated edges and the presence of pseudopodia. Compared with M1 cells, M2 macrophages exhibit a more regular morphology, with the disappearance of pseudopodia.

### 3.3 Volume Monitoring of Macrophages

#### 3.3.1 Volume monitoring of macrophages during cell death

**Fig. 3:**
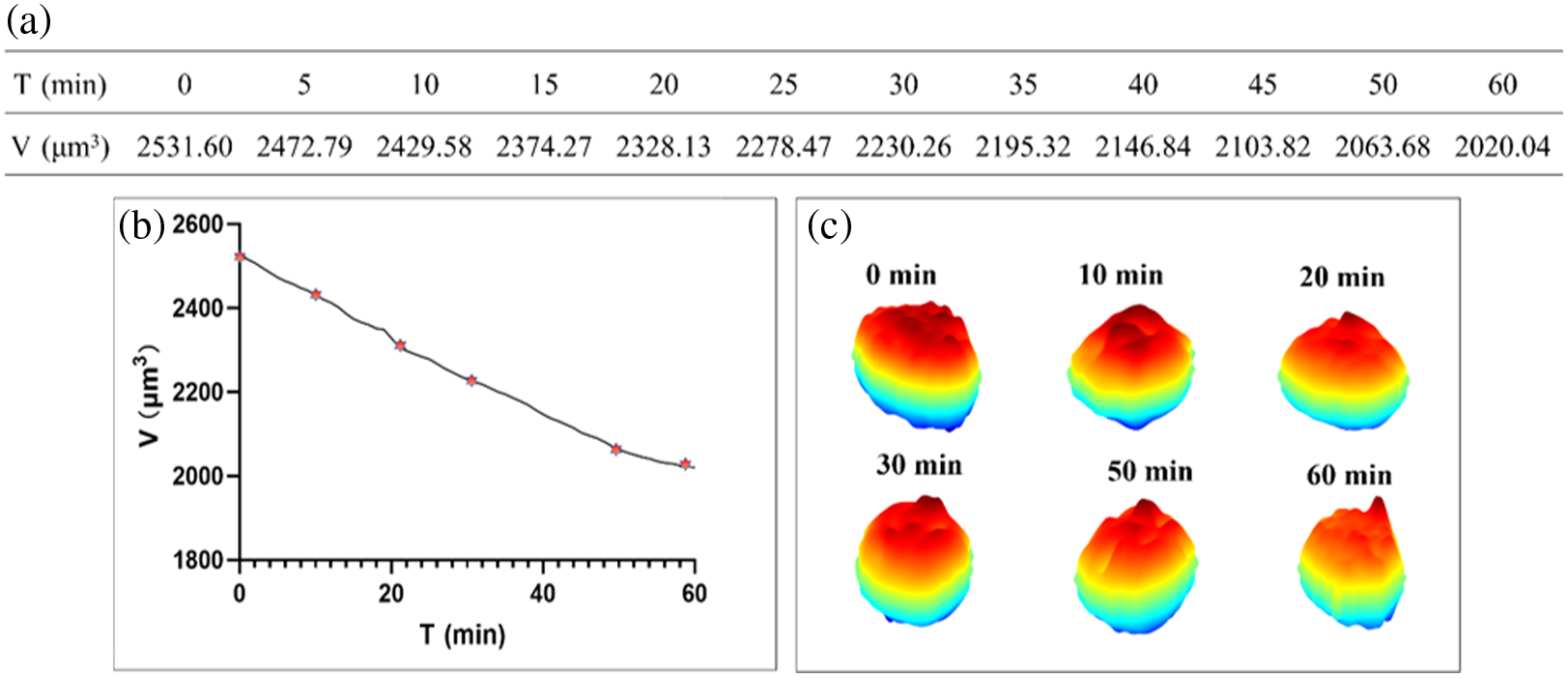
(a) Volumes of the M0 macrophage in 1 h (continuous tracking of N=1 representative cell). (b) Change of the volume during cell death. (c) 3D morphological distributions.

M0 macrophages were cultured in a dish with water and continuously monitored under hypotonic stress for a couple of hours. During this period, we observed a critical 1-h window where the cell exhibited a clear and rapid trend of morphological collapse, eventually detaching and floating in the culture medium. The volumetric variations within 1 h were calculated and listed in Fig. 3(a), and the corresponding volumetric evolution curve during cell death was plotted in Fig. 3(b). Figure 3(c) presents the 3D morphological distributions at six inflection points of the curve. At the initial state (0 min), the cell volume was $2531.60\;\mu m^{3}$. The phase distribution shows an intact cell morphology with a large thickness. When cell death began (15 min), the cell volume decreased to $2374.27\;\mu m^{3}$, and the effective phase height dropped significantly, indicating cell shrinkage. At 20 min, the volume further declined to $2328.13\;\mu m^{3}$, and local protrusions emerged in the phase image, implying rapid membrane remodeling characteristic of early cell death. From 30 to 50 min, the cell volume continued to fall from 2230.26 to $2063.68\;\mu m^{3}$, whereas the phase height gradually increased and became more uniform. This suggests that during this mid-stage volume reduction, substantial intracellular redistribution may be taking place. Finally, at 60 min, the cell volume reached its minimum value of $2020.04\;\mu m^{3}$, and the phase profile became smoother and more stable, indicating that the cell entered the late phase of cell death with diminished morphological dynamics.

#### 3.3.2 Volume monitoring of macrophages during cell division

**Fig. 4:**
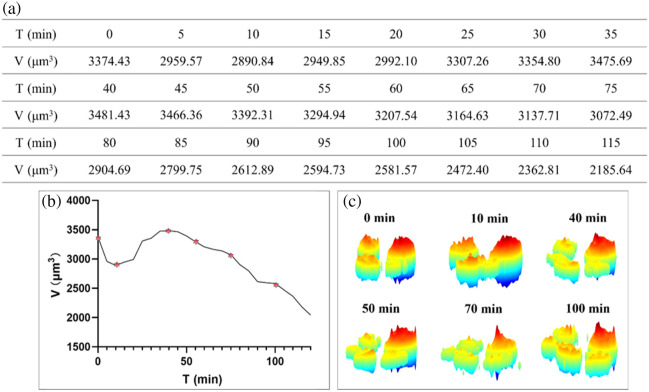
(a) Volumes of the M1 macrophage in 2 h (continuous tracking of N=1 representative cell). (b) Change of the volume during cell division. (c) 3D morphological distributions.

M1 macrophages were cultured in a dish containing growth medium to maintain their proliferative activity, and an interesting phenomenon was observed in which the M1 macrophages underwent cell division and cell death simultaneously. Figure 4(a) lists the volumetric data during this process, Fig. 4(b) shows the corresponding trend of volume variation, and Fig. 4(c) presents the 3D morphological distributions at the marked turning points of the curve. At the initial time, the cell volume was $3375.43\;\mu m^{3}$, with an intact phase peak. After 10 min, the volume decreased to $2890.84\;\mu m^{3}$, followed by an upward trend from 10 to 40 min, during which the volume rebounded and reached a peak of $3481.43\;\mu m^{3}$. The phase height at the top of the cell increased further, accompanied by more prominent protrusions, indicating enhanced intracellular activity and increased structural complexity. The phase distribution also shows an increase in cell number, confirming that cell division occurred during this stage and contributed to the volume increase. The overall trend from 40 to 120 min was a decline, and the 3D morphology shows a corresponding decrease in phase height, along with reduced cell volume and thickness, revealing structural loosening and a shift toward cell death. Meanwhile, two slight increases in volume were observed, suggesting that some intracellular activities were still taking place during these periods. At 120 min, the cell volume dropped sharply again to $2042.90\;\mu m^{3}$. The 3D morphology shows a corresponding decrease in phase height, along with reduced cell volume and thickness, revealing structural loosening and a shift toward death, eventually presenting a morphology with numerous spiky features.

It is important to clarify that the sharp volume increase observed during mitosis (e.g., peaking at $3481.43\;\mu m^{3}$) does not imply a violation of geometric volume conservation. Instead, this measurement represents the effective optical cluster volume of the dividing complex. During mitosis, macrophages round up and increase significantly in thickness, creating steep phase gradients that can induce phase halos in DHM. These optical artifacts can cause the segmentation algorithm to overestimate the cell boundary. Furthermore, the condensation of intracellular material locally increases the refractive index, which mathematically inflates the volume calculation under a constant-RI assumption. Thus, this peak serves as a distinct optical and morphological signature of the mitotic event rather than absolute geometric expansion.

#### 3.3.3 Volume monitoring of macrophages during the transition from M0 to M1

**Fig. 5:**
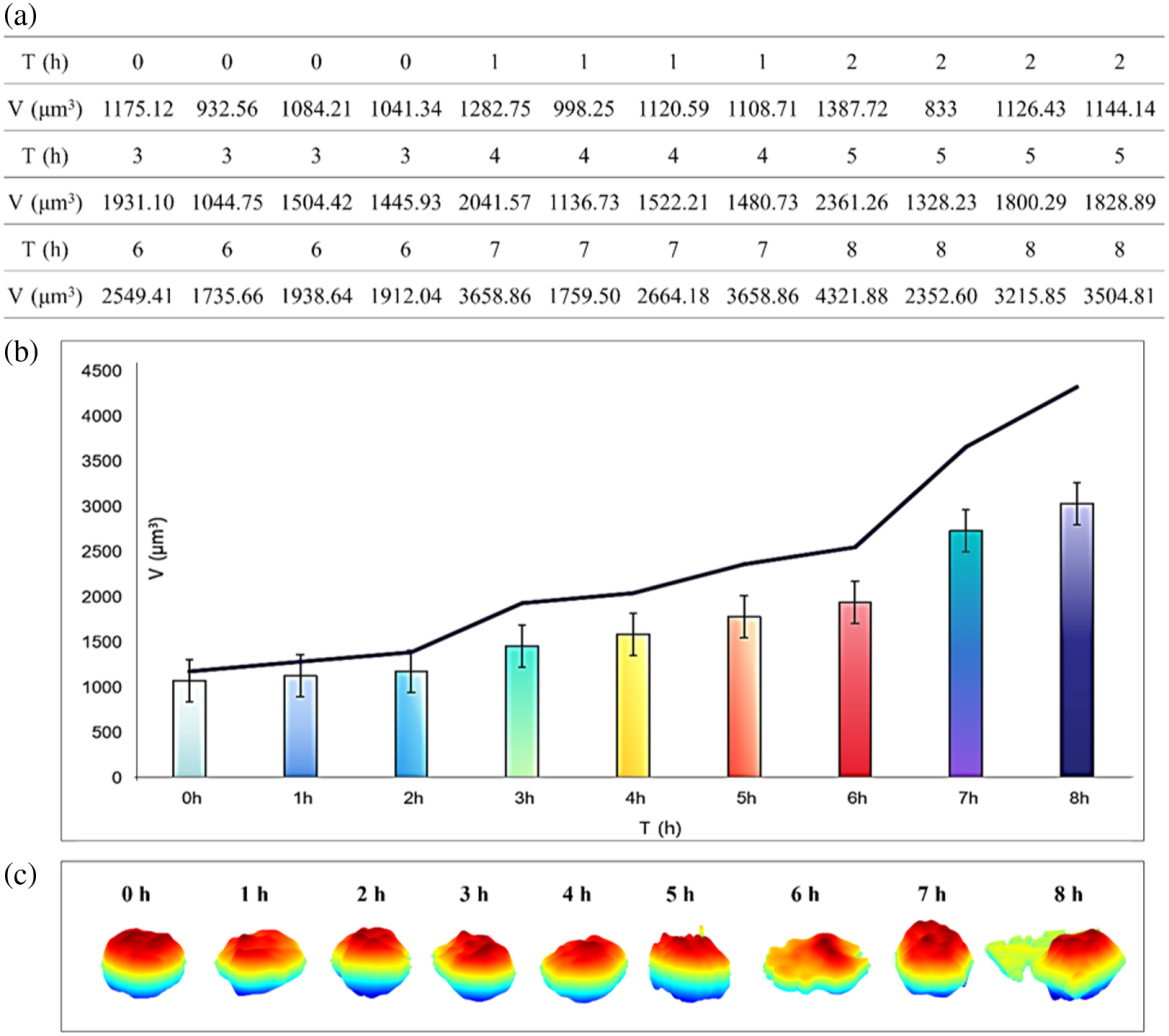
(a) Volumes within 8 h (sample sizes: *N* = 11, 19, 18, 26, 19, 21, 17, 11, and 10 cells for 0 to 8 h, respectively). (b) Change of the volume during the transition from M0 to M1. (c) 3D morphological distributions.

We monitored the volume changes of M0 macrophages induced with 200 ng/mL LPS during their transition to the M1 phenotype. The volumetric data, variation trends, and representative 3D morphologies are shown in Fig. 5. Figure 5(a) lists four representative volume values at each time point, including the maximum, minimum, and two intermediate values, the corresponding bar chart is shown in Fig. 5(b), and the volume evolution curve was plotted based on the maximum volume recorded in each hour. It can be found that the M0 to M1 polarization process involves four distinct phases, consisting of two relatively slow-growth stages and two rapid-growth stages. The slowest increase occurs during 0 to 2 h, indicating that M0 macrophages are not fully activated at the early stage but begin to exhibit activation tendencies, likely associated with metabolic activation. The steepest increase occurs during 6 to 8 h, suggesting strong cellular activation, substantial cytoskeletal remodeling, and a rapid shift toward the M1 phenotype.

For further analysis, from 0 to 2 h, the cell volume increases slowly from $1076.95\pm 85.27\;\mu m^{3}$ to $1126.96\pm 95.12\;\mu m^{3}$. From 2 to 3 h, the volume rises sharply to $1429.02\pm 265.40\;\mu m^{3}$. Between 3 and 6 h, the volume increase becomes moderate again, reaching $1977.20\pm 410.23\;\mu m^{3}$. From 6 to 8 h, the volume increases steeply once more, reaching a maximum of $3033.76\pm 787.14\;\mu m^{3}$. On average, the volume of M1 macrophages is approximately three times that of M0 macrophages. In terms of morphological changes, M0 macrophages exhibit a relatively smooth and rounded shape, whereas M1 macrophages display irregular shapes and even pseudopod-like protrusions.

## 4 Discussion and Conclusion

A limitation of this DHM-based single-wavelength measurement is the inherent coupling between the cell thickness and its intracellular refractive index (RI). In this study, cell volume was estimated assuming a constant RI difference (Δn). However, dynamic biological processes such as macrophage polarization and cell division are accompanied by substantial cytoskeletal remodeling, dry mass accumulation, and osmotic gradients, which inevitably cause local RI variations. In this work, the reported volume values represent the effective optical volume rather than the absolute geometric volume. The unquantified RI fluctuations introduce a degree of uncertainty into the absolute geometric measurements; for instance, the sharp volumetric increase observed during M1 polarization reflects a synergistic combination of true morphological expansion and an increase in intracellular optical density. In other words, even in scenarios where the absolute geometric volume of a cell remains relatively constant, intracellular structural and compositional remodeling induces local RI variations. Thus, the time-dependent changes in the measured optical volume can effectively reveal these macroscopic physiological dynamics. Nevertheless, as supported by previous quantitative phase imaging studies,^22^ relative changes in optical volume serve as a highly sensitive and robust label-free biomarker for capturing the biophysical signatures of cellular activation and state transitions without the need for complex RI-decoupling techniques.

In cell types beyond macrophages, numerous studies have also demonstrated nondestructive monitoring of cell volume holds significant value for elucidating underlying biological mechanisms, including ion transport, cytoskeletal reorganization, cell-cycle progression, and apoptosis. It may emerge as a sensitive indicator of cellular functionality.^23^^–^^27^ For example, corneal endothelial cells exhibit potassium-dependent swelling behavior that reflects their electrogenic transport capacity and graft viability.^23^ Likewise, epithelial cells under hypotonic challenge show characteristic swelling, blebbing, and eventual membrane rupture, reflecting the interplay between osmotic gradients and membrane stability. Aberrant volume regulation has also been implicated in pathological conditions. In glioma, the activity of the volume-regulatory transporter NKCC1 contributes to tumor progression and resistance to temozolomide, and its inhibition improves therapeutic outcomes by normalizing pathological cell swelling.^25^ In multidrug-resistant cancer cells, P-glycoprotein expression enhances the regulatory volume decrease response, linking volume control to drug-resistance mechanisms.^26^

In this work, the label-free and noninvasive DHM-based approach demonstrated provides a practical and effective means for quantifying cell volume without the need for exogenous contrast agents or physical perturbation. The ability to reconstruct 3D morphology from phase information enables continuous tracking of living cells over extended time periods. This is particularly advantageous for macrophages, with activation, polarization, and apoptotic processes that are accompanied by characteristic volume dynamics. The volume statistics of M0, M1, and M2 macrophages indicate that their volumes differ, thereby serving as an auxiliary means for assessing polarization states.

The volumetric profiles uncovered distinct slopes, fluctuation patterns, and inflection points, which might be the macroscopic manifestation of macrophage activation. The slow volume increase during the initial 0 to 2 h of LPS stimulation indicates a mild activation phase dominated by metabolic upregulation and early cytoskeletal reorganization. By contrast, the steep increase between 6 and 8 h represents a strong activation burst, likely associated with actin polymerization, pseudopod formation, and enhanced inflammatory functionality. Dying cells exhibited an opposite trend, showing a progressive decrease in volume accompanied by structural collapse. These results suggest that the rate of volume change (dV/dt) serves as a quantitative marker of macrophage activation strength, whereas the temporal pattern of volume evolution provides insight into the staging of the polarization process.

To overcome the current limitation of the inherent coupling between physical thickness and refractive index, future studies should consider incorporating tomographic schemes coupled to DHM (e.g., optical diffraction tomography). Although implementing such systems entails experimental challenges—particularly in configuring multi-angle vertical illuminations or sample rotation—it would grant access to genuinely three-dimensional maps of local intracellular refractive index (RI) variations. Alternatively, exploring hardware modifications such as dual-wavelength DHM or dual-medium decoupling techniques could also allow for the independent monitoring of dynamic RI changes. These technological extensions would eliminate the need to assume a constant refractive index, enabling the accurate extraction of absolute geometric volume and providing deeper biophysical insights into the intracellular structural remodeling of macrophages.

In our future work, we will further investigate the potential of using cell volume and its derived parameters—including the slope of volumetric change, fluctuation amplitude, and characteristic temporal milestones—as label-free biomarkers for evaluating immune status and pharmacological efficacy. Pharmacological interventions often alter cellular osmotic balance, cytoskeletal tension, and intracellular trafficking, all of which can manifest as distinct volumetric signatures. Quantifying these dynamics may offer a sensitive means of assessing drug responses. For example, the slope of volume change may correlate with the potency of anti-inflammatory agents, whereas the onset of major volumetric transitions may indicate the effective timescale of drug action.

## Data Availability

Code and data can be made available upon request to the corresponding author.
